# IORT for early-stage, low-risk breast cancer: Outcomes from a prospective, observational study

**DOI:** 10.1016/j.ctro.2025.100998

**Published:** 2025-06-22

**Authors:** Amalia Palacios-Eito, María del Carmen Moreno-Manzanaro, María Espinosa-Calvo, Fátima Ginés-Santiago, Juan Adrián Camús-Martínez, Ángel Calvo-Tudela, Pilar Rioja-Torres, Sara Romero-Martín, José Antonio Miñano-Herrero, Gustavo R. Sarria, Sonia García-Cabezas

**Affiliations:** a Department of Radiation Oncology, Reina Sofia University Hospital. Avda. Menéndez Pidal s/n, 14004 Cordoba, Spain; b Department of Radiation Oncology, Virgen de la Victoria University Hospital. Campus de Teatinos s/n 29010, Málaga, Spain; c Breast Surgery Unit, General and Digestive Surgery Department, Reina Sofia University Hospital. Avda. Menéndez Pidal s/n, 14004 Cordoba, Spain; d Breast Cancer Unit, Department of Diagnostic Radiology, Reina Sofía University Hospital. Avda. Menéndez Pidal s/n, 14004 Cordoba, Spain; e Department of Medical Physics, Reina Sofia University Hospital. Avda. Menéndez Pidal s/n, 14004 Cordoba, Spain; f Department of Radiation Oncology, University Hospital Bonn, Venusberg Campus 1, 53127 Bonn, Germany

**Keywords:** Intraoperative radiotherapy, Early-stage breast cancer, Accelerated partial breast irradiation, Real world

## Abstract

**Introduction:**

Treatment of early-stage, low-risk breast cancer (BC) has undergone significant de-escalation during the past years. The TARGIT-A trial provided information on intraoperative radiotherapy (IORT) as a convenient, single-fraction modality. Nevertheless, significant discussion regarding different aspects of the trial continues. This study reports on oncological outcomes in a real-world clinical setting and contributes to the understanding of its optimal indication.

**Methods:**

Five-hundred patients planned for breast-conserving surgery (BCS) were screened for IORT between June 2017 and December 2023, within this prospective, observational, single-center. The treatment protocol replicated the experimental arm of the TARGIT-A trial, implementing stricter inclusion criteria and maintaining a risk-adapted approach. The primary endpoint was ipsilateral breast recurrence rates (IBR). Secondary endpoints included local recurrence-free survival, progression-free survival, overall survival, and patient-reported cosmesis. The Kaplan-Meier method was used to analyze survival and recurrence risk, and risk factors for IBR were assessed through Cox regression analysis.

**Results:**

After screening and recruiting, IORT was performed in 464 patients. The median follow-up was 45.3 (8–89) months. The estimated 5-year IBR risk in the entire cohort was 1.7 % (95 % CI: 0.7 %–2.8 %), decreasing to 1 % (95 % CI: 0.3 %–2.4 %) in the IORT + whole breast irradiation (WBI) cohort. In the IORT-only cohort, the risk was 2.1 % (95 % CI: 0.6 %–3.7 %). No significant differences were observed among the three subgroups. The 5-year overall survival and breast cancer-specific survival rates were 97.6 % (95 % CI: 96.0 %–99.1 %) and 99.5 % (95 % CI: 98.5 %–100 %), respectively. Two independent significant risk factors for IBR were identified: age < 50 years (HR = 0.138, 95 % CI: 0.032–0.597, p = 0.008) and close or affected surgical margins (HR = 5.8, 95 % CI: 1.5–22.5, p = 0.011). No grade 3–4 toxicity events were reported. Patient-reported cosmesis was excellent/good in 84 % of cases.

**Conclusions:**

Local recurrence rates were low amongst all groups. Superior control outcomes could be obtained by applying more restrictive criteria than the TARGIT A trial. Longer follow-up is needed to confirm our findings.

## Highlights

• Refined criteria for IORT could benefit selected patients with breast cancer.
• In-breast recurrence ranges from 2.1% to 0.5% based on applied criteria.
• No severe adverse events were observed.
• Cosmesis was reported good or excellent by 84% of patients.

## Introduction

In recent years, local treatment for early-stage breast cancer has undergone significant therapeutic de-escalation, with a reduction in overall the number of mastectomies and axillary dissections, the adoption of hypofractionated irradiation schemes, and the implementation of accelerated partial breast irradiation (APBI) [1]. APBI has been established as an effective alternative to external whole-breast irradiation (EBRT), supported by multiple studies demonstrating comparable rates of ipsilateral breast recurrence (IBR) and favorable toxicity profiles [2].

In this context, IORT has emerged as a modality of APBI, allowing the administration of a concentrated radiation dose to the bed immediately after surgical resection. This technique provides maximum precision due to the direct visualization of the tumor bed and minimizes exposure to surrounding healthy tissues. Additionally, by being performed in a single procedure along with breast-conserving surgery, it reduces the healthcare burden for both the patient and the healthcare system [1]. The TARGIT-A trial, a randomized non-inferiority study comparing kilovoltage (kV) IORT with EBRT in early-stage breast cancer. The primary objective of TARGIT-A was the absolute difference in IBR between patients treated with IORT and those who received EBRT, establishing a non-inferiority margin of 2.5 %. This trial implemented a risk-adapted approach, wherein patients with pathological risk factors received additional whole-breast irradiation (WBI). At five years, the IBR rate was 2.11 % with IORT versus 0.95 % with EBRT [3]. In accordance with the risk adapted approach, 20 % of patients subsequently required WBI due to the presence of high-risk factors. An update reporting at 8.6 years showed no differences in local recurrence-free survival, mastectomy-free survival, distant disease-free survival, overall survival nor breast cancer mortality [3].

Nevertheless, criticism on certain points, including design and reporting, have questioned the validity of these outcomes [4]. Given the balance between benefits and risks, it is essential to establish strict patient selection criteria within risk-adapted frameworks. The present study aims to provide real-world data, contributing to select patients who could benefit from IORT as PBI, offering prospective data to further support the efficacy and safety of kV IORT.

## Materials and methods

### Study Design and Population

IORT using a 50 kV photon device (INTRABEAM®, Carl Zeiss Meditec, Oberkochen, Germany) was implemented in our department in June 2017. A prospective observational study was conducted at our tertiary referral center, expecting 500 patients with early-stage, low-risk breast cancer to be screened. All cases were discussed at the multidisciplinary breast tumor board. This study was approved by the Institutional Ethics Committee (protocol code ref. 3879, Acta n° 276), adhering to the principles of the Declaration of Helsinki and national regulations. All patients meeting the predefined selection criteria were included from the study initiation date. A risk-adapted approach was employed, with the addition of WBI in patients with high-risk pathological findings.

In our analysis, the primary endpoint was ipsilateral breast recurrence. Secondary endpoints included local recurrence-free survival, progression-free survival, overall survival, and patient-reported cosmesis.

**Selection criteria** were based on the experimental arm of the TARGIT-A trial with additional restrictions derived from available evidence at the time. Inclusion criteria consisted of age ≥ 45 years, tumors ≤ 2.5 cm (with a surgical cavity ≤ 5 cm), unifocal invasive ductal carcinoma, histological grade G1-G2, no extensive intraductal component (<25 %), positive hormone receptors (ER + with or without PR + ) and HER2-negative status, and no clinical or ultrasound evidence of nodal involvement. Patients with pure ductal carcinoma in situ (DCIS) were not included.

**Exclusion criteria** comprised an extensive intraductal component (>25 %), extensive lymphovascular or perineural invasion, lobular histology, histological grade G3, nodal involvement (isolated tumor cells, N1mic, N1, N2-N3), male patients (previously excluded in randomized clinical trials), and patients who had received neoadjuvant chemotherapy.

### Therapeutic Procedure

The diagnostic procedure included digital mammography with tomosynthesis, breast and axillary ultrasound, contrast-enhanced breast MRI or contrast mammography when MRI was not feasible, and core needle biopsy for histological confirmation.

Axillary nodal involvement was excluded through a negative preoperative axillary ultrasound. Surgical margins were assessed via mammographic imaging of the excised specimen. When the radiologist reported close margins, an intraoperative re-excision was performed.

All patients received a single dose of 20 Gy prescribed to the applicator surface. When necessary, WBI was delivered postoperatively or after systemic chemotherapy using a moderately hypofractionated regimen of 2.67 Gy in 15 fractions to a total dose of 40.05 Gy.

During surgery, additional exclusion criteria for IORT were applied. These included insufficient coverage of the surgical cavity due to size or shape, inadequate breast tissue coverage of the applicator (such as in inner quadrants, near the inframammary fold, or in small breasts), and failure to identify the sentinel lymph node intraoperatively. Surgical margins < 2 mm was considered close in the histology report.

WBI was additionally indicated in cases of surgical margins < 1 mm, extensive intraductal component (>25 %) (assessed individually based on the proportion of DCIS and invasive ductal carcinoma), unexpected lobular carcinoma histology, histological grade G3, nodal involvement (isolated tumor cells, N1mic, N1, N2-N3), or extensive lymphovascular or perineural invasion.

All patients received adjuvant endocrine therapy unless contraindicated due to comorbidities. Adjuvant chemotherapy was administered in cases presenting high-risk features, either based on pathological findings or genomic risk scores.

### Follow-Up and Evaluation

Patients were examined one month after completing radiotherapy and then annually thereafter. Follow-up visits involved documentation of postoperative complications, acute and chronic toxicity assessed using the **NCI CTCAE** V.4 scale [5], IBR and a cosmetic evaluation. Acute toxicity was assessed one month post-treatment, regardless of whether patients received IORT alone or IORT combined with WBI. Late toxicity was evaluated at 12 months following the end of all radiotherapy.

**Cosmetic outcomes** were assessed according to the patient-reported classification system for cosmetic results after breast-conserving therapy established by the European Organization for Research and Treatment of Cancer (EORTC) [6]. Patients were asked to compare their treated breast with the untreated one and to rate specific aspects, including breast size and shape, areola/nipple position and shape, skin color, breast edema, surgical scar appearance, telangiectasia, and overall cosmetic outcome. The classification used a four-point scale: no difference (excellent), slight difference (good), moderate difference (fair), and significant difference (poor). Cosmetic evaluation was based on a single patient-reported outcome collected during the 12-month post-treatment visit.

### Statistical analysis

Baseline characteristics were analyzed using descriptive statistics. Local control and survival rates were estimated using the Kaplan-Meier method, and comparisons were made using the log-rank test. IBR was defined as any in situ or invasive event in any quadrant of the treated breast. The time to relapse was the interval between surgery and the diagnosis of recurrence. Five-year IBR risk was estimated by calculating the hazard function. A multivariate Cox regression analysis was subsequently conducted to evaluate the risk factors associated with IBR, including the following variables: age (<50 years vs. ≥ 50 years), pT stage, pN status, differentiation grade, lymphovascular invasion, presence of DCIS, close and/or affected margins, and the administration of WBI. A p-value < 0.05 was considered statistically significant. SPSS 27® software (IBM, US) was used for all statistical analyses.

For patients treated with exclusive IORT, a simulation was conducted applying the recommended criteria from two PBI consensus guidelines (American Society for Radiation Oncology ASTRO 2009[7] and Groupe Européen de Curietherapie–European Society for Radiotherapy GEC-ESTRO 2009[8]), as well as the inclusion criteria of the TARGIT-A trial and the excellent prognosis subgroup described by Whelan[9], to determine recurrence rates in homogeneous cohorts. The hazard function in each subgroup was calculated to estimate the simulated 5-year IBR risk.

## Results

### Study Population

Between June 2017 and December 2023, a total of 500 patients with early-stage breast cancer were screened and recruited in the study. In 36 patients (7 %), IORT was not performed intraoperatively due to patient-specific anatomical constraints or surgical considerations**.** In the remaining 464 patients, the procedure was performed.

The patient and tumor features are listed in Table 1. The causes for IORT cancellation during surgery and the applicator diameters used are summarized in Table 2.In 133 patients (28.7 %), WBI was administered due to high-risk pathological findings, leaving a total of 331 patients (71.3 %) in the exclusive IORT group.

**Table 1:** Patient and treatment characteristics.

**Patient and treatment characteristics**	
Characteristics	N (%)
No of patients	464 (100 %)
Age (years) Median (Range)	60 (45–82)
Breast laterality	
Right	217 (46.8 %)
Left	247 (53.2 %)
Tumor Location	
Upper Outer Quadrant	166 (35.8 %)
Upper Inner Quadrant	75 (16.2 %)
Lower Outer Quadrant	20 (4.3 %)
Lower Inner Quadrant	34 (7.3 %)
Retroareolar	12 (2.6 %)
Outer Quadrant Transition	45 (9.7 %)
Upper Quadrant Transition	71 (15.3 %)
Lower Quadrant Transition	30 (6.5 %)
Inner Quadrant Transition	11 (2.4 %)
Tumor size cT	
cT1a	12 (2.6 %)
cT1b	178 (38.4 %)
cT1c	243 (52.4 %)
cT2	31 (6.7 %)
Molecular classification	
Luminal A	407 (87.7 %)
Luminal B	57 (12.3 %)
Tumor size pT	
pT0	1 (0.2 %)
pTis	2 (0.4 %)
pT1a	34 (7.3 %)
pT1b	155 (33.4 %)
pT1c	231 (49.8 %)
pT2	41 (8.8 %)
pN	
pN0	391 (84.3 %)
pN0i+	6 (1.3 %)
pN1mi	23 (5 %)
pN1a	35 (7.5 %)
pN2a	1 (0.2 %)
pNx	8 (1.7 %)
Grade	
G1	267 (57.5 %)
G2	184 (39.7 %)
G3	13 (2.8 %)
LVI	
No	434 (93.5 %)
Yes	17 (3.7 %)
Focal	2 (0.4 %)
Inconclusive	11 (2.4 %)
CDIS	
No	213 (46 %)
Yes (<25 %)Yes (≥25 %)	184 (39.6 %)67 (14.4 %)
Pathologic margins	
Negative	411 (88.6 %)
Close (< 2 mm)	30 (6.5 %)
Positive	23 (5 %)
Re-excision	
No	427 (92 %)
Yes	37 (8 %)
Adjuvant chemotherapy	
Yes	50 (10.8 %)
No	414 (89.2 %)
Hormone therapy	
Yes	441 (95 %)
No	23 (5 %)

**Table 2:** Diameters of the applicator used and reasons for the rejection of IORT intra-procedure.

Applicator diameter (N = 464)	Reasons for the rejection of IORT (N = 36)	N (%)
3.5 cm (54.2 %)	Irregular / unsuitable bed	16 (44.4 %)
4 cm (21.2 %)	Proximity to skin	14 (38.9 %)
3 cm (10.6 %)	No identification sentinel lymph node	2 (5.6 %)
4.5 cm (4.6 %)	Hemodynamic instability	2 (5.6 %)
5 cm (2 %)	Sentinel lymph node suspected of malignancy	1(2.8 %)
2.5 cm (0.2 %)	Tumor growth > 2.5 cm	1 (2.8 %)

#### Reasons for WBI administration

The main reasons for WBI administration were nodal involvement in 64 patients (48.1 %), an extensive intraductal component of ≥ 25 % in 27 patients (20.3 %), and surgical margins < 1 mm in 13 patients (9.8 %). Other indications included tumors classified as grade 3 (10 patients, 7.5 %), lobular or mixed histology (4 patients, 3 %), luminal B molecular subtype (6 patients, 4.5 %), HER2 + status (4 patients, 3 %), pNx classification (4 patients, 3 %), and extensive lymphovascular invasion (1 patient, 0.8 %). Of the 133 patients who received WBI, 44 had also received adjuvant chemotherapy.

#### Local recurrence analysis

The median follow-up was 45.3 months (range: 8–89 months).

There were 12 ipsilateral breast recurrences: 10 in the exclusive IORT group (10/331), of which 7 occurred in the same quadrant (4 in the upper outer quadrant, 1 retroareolar, 1 in the upper inner quadrant, and 1 in the upper quadrant transition) and 3 in different quadrants (1 in the upper outer quadrant, 1 in the outer quadrant transition and 1 in the inner quadrant transition); and 2 in the IORT + WBI group (2/133), 1 in the same quadrant (upper outer quadrant) and 1 in a different quadrant (upper quadrant transition).

No local recurrences were detected in the remaining 36 patients in whom IORT was not performed.

A total of 11 deaths (2.2 %) were reported during follow-up, of which 9 were unrelated to breast cancer and 2 were due to metastatic progression. The estimated 5-year IBR risk for the entire cohort was 1.7 % (95 % CI: 0.7 %–2.8 %). In the exclusive IORT cohort, the risk was 2.1 % (95 % CI: 0.6 %–3.7 %), whereas in the IORT + WBI cohort, it was 1 % (95 % CI: 0.3 %–2.4 %).

The median time to recurrence was 37.4 months (range: 12.4–60.6 months). The histology of recurrence was infiltrative carcinoma in 11 cases (91.7 %) and ductal carcinoma in situ in 1 case (8.3 %). The recurrence location was within the initial tumor bed (in-field recurrence) in 8 cases (66.7 %), while 4 cases (33.3 %) occurred in a different quadrant of the treated breast.

A total of 11 patients underwent reoperation, with 6 patients (54.5 %) requiring mastectomy and 5 (45.5 %) undergoing a second lumpectomy. One patient with local recurrence was initially scheduled for mastectomy but developed distant metastases before surgery and was subsequently treated with chemotherapy. All patients who underwent a second lumpectomy received WBI. No second local recurrences or deaths due to locoregional relapse were recorded. The characteristics of patients with local recurrence are presented in Table 3.

**Table 3:** Characteristics of patients with local recurrence.

N	Age	Stage	Grade	Molecular classification	RT	Pathologic margins	LVI	DCIS (%)	CT	HT	Time-to-recurrence (mo)	Quadrant	Treatment of recurrence	Histology of recurrence
1	60	pT1cN0	G2	Luminal A	IORT	Negative	No	No	No	Yes	60.6	Other	Mastectomy	IDC
2	49	pT1cN0	G1	Luminal A	IORT	Negative	No	No	No	Yes	36.7	Other	Lumpectomy	IDC
3	63	pT1cN0	G1	Luminal A	IORT	Negative	No	Yes (<25 %)	No	Yes	27.5	Same	Lumpectomy	IDC
4	46	pT1cN0	G2	Luminal A	IORT	Negative	No	Yes (<25 %)	No	Yes	38.3	Same	Mastectomy	IDC
5	57	pT1cN0	G2	Luminal B	IORT	Close (R)	No	Yes (<25 %)	No	Yes	38.1	Same	Lumpectomy	IDC
6	58	pT1cN0	G1	Luminal A	IORT	Negative	No	No	No	No	58.6	Same	Mastectomy	IDC
7	62	pT1cN0	G1	Luminal A	IORT + WBI	Close (R)	No	Yes (<25 %)	No	Yes	45.6	Same	Mastectomy	IDC
8	54	pT1bN1mi	G2	Luminal A	IORT + WBI	Close (R)	Inconclusive	Yes (>25 %)	No	Yes	24.0	Other	Mastectomy	DCIS
9	57	pT1N0	G1	Luminal A	IORT	Negative	No	Yes (<25 %)	No	Yes	12.4	Same	Chemotherapy	IDC
10	58	pT1cN0	G2	Luminal B	IORT	Close (NR)	No	No	No	Yes	36.4	Other	Lumpectomy	IDC
11	48	pT1cN0	G2	Luminal A	IORT	Negative	No	Yes (<25 %)	No	Yes	35.9	Same	Lumpectomy	IDC
12	63	pT0N0	G1	Luminal A	IORT	Negative	No	No	No	Yes	55.5	Same	Mastectomy	IDC

RT, radiation therapy; IORT, intraoperative radiation therapy; LVI, lymphovascular invasion; WBI, whole-breast irradiation; DCIS, ductal carcinoma in situ; CT, chemotherapy; HT, hormone therapy. IDC, infiltrating ductal carcinoma. R: Re-excision. NR: No re-excision.

#### Factors associated with IBR risk

In the multivariate Cox proportional hazards model analysis, two independent predictors of IBR were identified. Patients aged ≥ 50 years had a significantly lower risk of recurrence compared to those < 50 years (HR = 0.138; 95 % CI: 0.032–0.597; p = 0.008). Close or affected surgical margins were also associated with a significantly increased risk of recurrence (HR = 5.8; 95 % CI: 1.5–22.5; p = 0.011). The results of the survival analysis are summarized in Table 4.

**Table 4:** Five-years Kaplan-Meier estimates of results for the IORT population, for the exclusive IORT group, and for the IORT + WBI group.

Five-year results	N = 494	N = 331	N = 133
Kaplan-Meier estimates (95 %CI)	IORT	Exclusive IORT	IORT + WBI
Local recurrence free-survival	96 % (93.4 % − 98.5 %)	95.2 % (91.8 % − 98.5 %)	97.7 % (94.5 %-100 %)
Mastectomy free survival	98.1 % (96.3 % − 99.8 %)	98.2 % (96 % − 100 %)	97.7 % (94.3 %-100 %)
Distant recurrence-free survival	99.4 % (98 % − 100 %)	99.7 % (99.1 % − 100 %)	98.5 % (96.1 %-100 %)
Recurrence and/or progression-free survival	95 % (92.2 % − 97.7 %)	94.3 % (90.6 % − 98 %)	96.4 % (92.3 %-100 %)
Overall survival	97.7 % (96.1 % − 99.2 %)	97.8 % (96 % − 99.5 %)	97.5 % (94 %-100 %)
Cancer-specific overall survival	99.5 % (98.5 % − 100 %)	100 %	98.4 % (95.2 %-100 %)

#### Toxicity and cosmetic evaluation

**Fig. 1:**
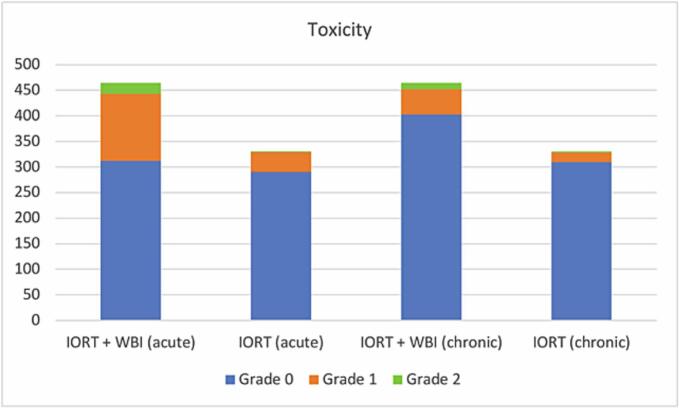

Regarding acute toxicity, grade 0 was observed in 312 patients (67.2 %), grade 1 in 131 patients (28.2 %), and grade 2 in 21 patients (4.5 %). Chronic toxicity was classified as grade 0 in 403 patients (86.9 %), grade 1 in 49 patients (10.6 %), and grade 2 in 12 patients (2.6 %). No grade 3 or higher adverse events were reported. In Fig. 1, the toxicity is shown based on whether the treatment was performed with IORT + WBI or with exclusive IORT.

The patient-reported cosmetic evaluation showed excellent results in 51.3 % of cases, good in 33.2 %, fair in 13.6 %, poor in 1.7 %, and unknown in 0.2 %. The 15.3 % (regular and poor results) was reduced to 6.3 % in the exclusive IORT group.

Among the 37 patients reoperated for margin enlargement, those who also received WBI had significantly worse cosmetic results (Excellent/Good: 52.6 %) compared to those who did not receive WBI (Excellent/Good: 94.5 %), p = 0.016.

## Discussion

The estimated risk of IBR in our series (1.7 % at five years) was comparable or slightly lower than that reported in the pivotal TARGIT-A trial (2.11 % at five years), which could be related to stricter selection criteria in our series. Likewise, our results are consistent with those of other published series, supporting the safety and efficacy of kV IORT within a risk-adapted approach. Vinante et al. [10], in a study of 814 patients, reported an IBR of 2.5 % (95 % CI: 1.7 %-3.3 %). Tallet et al. [11], in their analysis of 676 patients, described an IBR of 1.7 %. Similarly, Mi et al.[12], in a cohort of 128 patients, documented an IBR of 2.3 %. Silverstein et al.[13] described an overall IBR probability of 5.18 % in a cohort of 1,600 patients treated with IORT, although they identified a subgroup with favorable characteristics in which the IBR risk at five years was reduced to 1.83 %.

The IBR rate observed in our series (1.7 %) is comparable to those reported in recent trials using ultrahypofractionated WBI. For instance, the FAST-Forward trial[14] reported 1.4 % IBR at 5 years for the 26 Gy/5 fractions group, and 1.7 % for the 27 Gy/5 fractions group, with median follow-up around 62 months. Systemic therapies may have contributed to the outcomes observed in this and other studies involving similar low-risk early-stage breast cancer populations. The vast majority of patients received endocrine therapy, and chemotherapy was administered in high-risk cases, both of which are known to improve local control and survival.

The median follow-up in our study (45.3 months) is similar or slightly shorter than in other series with comparable IBR rates, such as Tallet [11](54 months, IBR 1.7 %), Silverstein [13](62 months, IBR 1.83 %), and Vinante[10] (72 months, IBR 2.5 %).

While recent trials have explored the omission of radiotherapy in low-risk patients (e.g., LUMINA[15], PRIME II[16], CALGB 9343[17]), their IBR rates remain higher than those observed with IORT. Moreover, a SEER-based analysis[18] showed that IORT was associated with significantly better 5-year breast cancer-specific survival compared to omission of EBRT (99.3 % vs. 97.5 %).

To highlight the importance of selection criteria, we conducted a simulation analysis to estimate the IBR risk that would have been observed in our cohort treated exclusively with IORT if we had only included cases meeting the selection criteria recommended by GEC-ESTRO [8], ASTRO [19], TARGIT-A [20], and the excellent prognosis subgroup described by Whelan [9]. The results showed a five-year IBR range between 2.1 % and 0.5 %, with the highest local control achieved when applying Whelan (0.5 %) and ASTRO (1.4 %) criteria (Table 5). These findings support the need for more restrictive patient selection to optimize clinical outcomes.

**Table 5:** IBR risk simulation over 5-years based on different criteria used for IORT.

Characteristics	Our Criteria	ASTRO Criteria	GEC-ESTRO Criteria	TARGIT A Criteria	Whelan Criteria
	N = 331	N = 294	N = 307	N = 331	N = 169
Age (years)	≥45	≥60	≥50	≥45	≥55
Tumor size (cm)	<2.5	≤2	≤3	<3.5	≤2
Tumor grade	G1-2	Any	Any	Any	G1-2
Histology	IDC	IDC	IDC	IDC	IDC
Ductal in situ carcinoma only	No	No	No	No	No
Node status	pN0	pN0	pN0	cN0-1	pN0
Margins	≥1mm	≥2mm	≥2mm	≥1mm	≥1mm
Lymphovascular invasion	No	No	No	No	No
Estrogen receptor	Positive	Positive	Any	Any	Positive
HER2 overexpression	No	No	No	Any	No
5-years IBR risk (95 % CI)	2.1 % (0.6 % − 3.7 %)	1.4 % (0.13 % − 2.7 %)	1.6 % (0.2 % − 3 %)	2.1 % (0.6 % − 3.7 %)	0.5 % (0.2 % − 1.3 %)

IDC, infiltrating ductal carcinoma.

Various clinical and biological factors associated with age make it a key criterion in all PBI studies, as it correlates with local recurrence risk. In our series, patients aged 50 years or older had an 86.2 % lower IBR risk than those under 50 years. This finding encourages us to refine our selection criteria for prescribing IORT. This criterion is already incorporated into ASTRO (≥60 years), GEC-ESTRO (≥50 years) and the Spanish Consensus (≥60 years or ≥ 50 years in postmenopausal women) recommendations [21].

In most randomized trials comparing PBI versus EBRT, positive surgical margins were an exclusion criterion. However, the two randomized trials of IORT versus EBRT included patients with positive margins, albeit at a very low rate: <1% in the ELIOT trial and 6 % in the TARGIT trial, where WBI was associated in these cases. In our series, we found that a close (<2 mm) or positive margin was associated with an HR = 5.8 for IBR, meaning that our patients with insufficient or positive margins had nearly six times the probability of developing IBR compared to those with clear margins. Although a margin re-excision was performed in most of our patients with positive margins (87 %) and in some of those considered to have close margins (46.6 %), our current protocol only considers WBI for margins < 1 mm, regardless of previous margin re-excision. This observation leads us to reconsider or expand our criteria for when to associate WBI based on margin status, which could significantly impact reducing IBR rates.

The overall IBR risk in our series was 1.7 %, which was reduced to 1 % in patients who received complementary WBI due to risk factors identified in the pathological examination. This reduction is consistent with most published series [3]; Vinante et al [22]; Martínez et al. [23]. In the TARGIT-A trial, this risk was reduced to 0.9 % (95 % CI: 0.1–6.1) [20]. Although these events are rare and do not impact survival [3,18,24], the ASTRO 2024 clinical practice guideline[1] does not recommend PBI with kV IORT as a standalone modality without WBI, except in regulated clinical trials.

Although recent ASTRO guidelines categorize IORT as investigational outside of clinical trials or registries, our study was conducted within the framework of an approved prospective institutional protocol with ethics committee approval. In this context, the use of IORT was justified based on patient selection aligned with published criteria, as well as the objective to generate real-world evidence.

In our series, 26.6 % of patients received complementary WBI after postoperative pathological analysis. In the TARGIT-A trial, this proportion was 21.6 % in the prepathological cohort [25], aligning with our findings. However, the reported WBI completion rate varies widely in the literature: Li et al. [26] reported 61.3 %, Salari et al. [27] 13 %, Andraos et al. [28] 13.8 %, Brown et al. [29] 17.3 %, Tallet et al. [11] 31 %, Vinante et al. [10] 39.7 %, and Mi et al. [12] 6.1 %. This variability arises from differences in initial selection criteria and pathological factors considered for recommending additional WBI [30].

The volume of irradiated tissue varies depending on the PBI technique. In brachytherapy, the target includes the tumor bed plus 2.0 cm [31,32]. In 3D-CRT, the irradiated volume consists of the tumor bed with a 1–1.5 cm margin and an additional planning target volume (PTV) of 0.5–1 cm [[33], [34], [35], [36]]. In TARGIT-A and in our series, the prescription was 20 Gy at the tumor bed surface, with 5–7 Gy at 1 cm depth [3]. This smaller irradiated volume with kV IORT correlates with a proportional reduction in unwanted side effects [[37], [38], [39]].

Cosmetic outcomes were influenced not only by radiation toxicity but also by the need for re-excision and subsequent WBI. Patients who required both re-excision and WBI showed significantly poorer aesthetic results compared to those re-excised but treated with IORT alone. This suggests that the combination of surgical reintervention and extended irradiation volumes may negatively impact cosmetic perception.

The main limitation of this study is its median follow-up of 45.3 months, a relatively short period for assessing long-term local recurrence risk [3,10,40]. In addition to the follow-up duration, the absence of a randomized control group limits the ability to compare this strategy with standard WBI or omission of irradiation. Nevertheless, the design reflects real-world applicability.

In the current context of therapeutic de-escalation, physicians face the challenge of balancing treatment reduction—minimizing side effects and improving quality of life—while maintaining adequate disease control. Kv IORT is an alternative in this scenario, offering both clinical and socioeconomic advantages. It ensures 100 % therapeutic adherence, reduces the perceived severity of the disease, and minimizes late toxicity by decreasing radiation exposure to the skin, heart, and lungs. IORT has been associated with lower non–breast cancer mortality, as reported in the long-term results of the TARGIT-A trial[3], which observed fewer deaths unrelated to breast cancer—including cardiovascular and other causes—in the IORT arm compared to standard whole-breast irradiation.

Additionally, the aesthetic outcome is highly satisfactory [38,[41], [42], [43]] and the ability to complete treatment in a single surgical procedure enhances the physical and psychosocial well-being of patients [37].

From a healthcare system perspective, IORT offers multiple advantages. It is particularly suitable for patients with reduced mobility, neurodegenerative diseases such as Parkinson’s disease and multiple sclerosis, or psychiatric disorders [44,45], where proper immobilization is challenging. Its implementation results in cost savings for both patients and the healthcare system [46,47].

In a setting where IORT provides significant benefits for both patients and the healthcare system, with a slight increase in IBR risk that does not impact survival, it is essential to provide patients with detailed and balanced information on its advantages and limitations, allowing them to make an informed treatment decision. To this end, the National Institute for Health and Care Excellence (NICE) has published a document on its website analyzing the pros and cons of Kv IORT, providing a decision-making tool for both patients and healthcare professionals [48].

## Conclusions

Local recurrence rates were low amongst all groups. Superior control outcomes could be obtained by applying more restrictive criteria than the TARGIT A trial. Excellent cosmesis outcomes were consistently reported by most patients. Longer follow-up is needed to confirm our findings.

## CRediT authorship contribution statement

**Amalia Palacios-Eito:** Conceptualization, Data curation, Writing – original draft. **María del Carmen Moreno-Manzanaro:** Data curation, Writing – original draft. **María Espinosa-Calvo:** Investigation. **Fátima Ginés-Santiago:** Methodology. **Juan Adrián Camús-Martínez:** Investigation. **Ángel Calvo-Tudela:** Conceptualization. **Pilar Rioja-Torres:** Resources. **Sara Romero-Martín:** Visualization. **José Antonio Miñano-Herrero:** Validation. **Gustavo R. Sarria:** Conceptualization, Supervision, Writing – review & editing. **Sonia García-Cabezas:** Conceptualization, Supervision, Writing – review & editing, Data curation, Formal analysis.

## Declaration of Competing Interest

The authors declare that they have no known competing financial interests or personal relationships that could have appeared to influence the work reported in this paper.

Amalia Palacios-Eito: nothing to declare.

María del Carmen Moreno-Manzanaro: nothing to declare.

María Espinosa-Calvo: nothing to declare.

Fátima Ginés-Santiago: nothing to declare.

Juan Adrián Camús-Martínez: nothing to declare.

Ángel Calvo-Tudela: nothing to declare.

Pilar Rioja-Torres: nothing to declare.

Sara Romero-Martín: nothing to declare.

José Antonio Miñano-Herrero: nothing to declare.

Gustavo R. Sarria: grants, travel costs and personal fees from Carl Zeiss Meditec AG, not related to this work.

Sonia García-Cabezas: nothing to declare.
